# Supplementary material for: Nanostructuring one-dimensional and amorphous lithium peroxide for high round-trip efficiency in lithium-oxygen batteries
Source: Nat Commun. 2018 Feb 14;9:680. doi: 10.1038/s41467-017-02727-2 (PMC5813182; doi:10.1038/s41467-017-02727-2)
Supplement: Supplementary file 1 — Supplementary Information [file 41467_2017_2727_MOESM1_ESM.pdf]

Supplementary Information

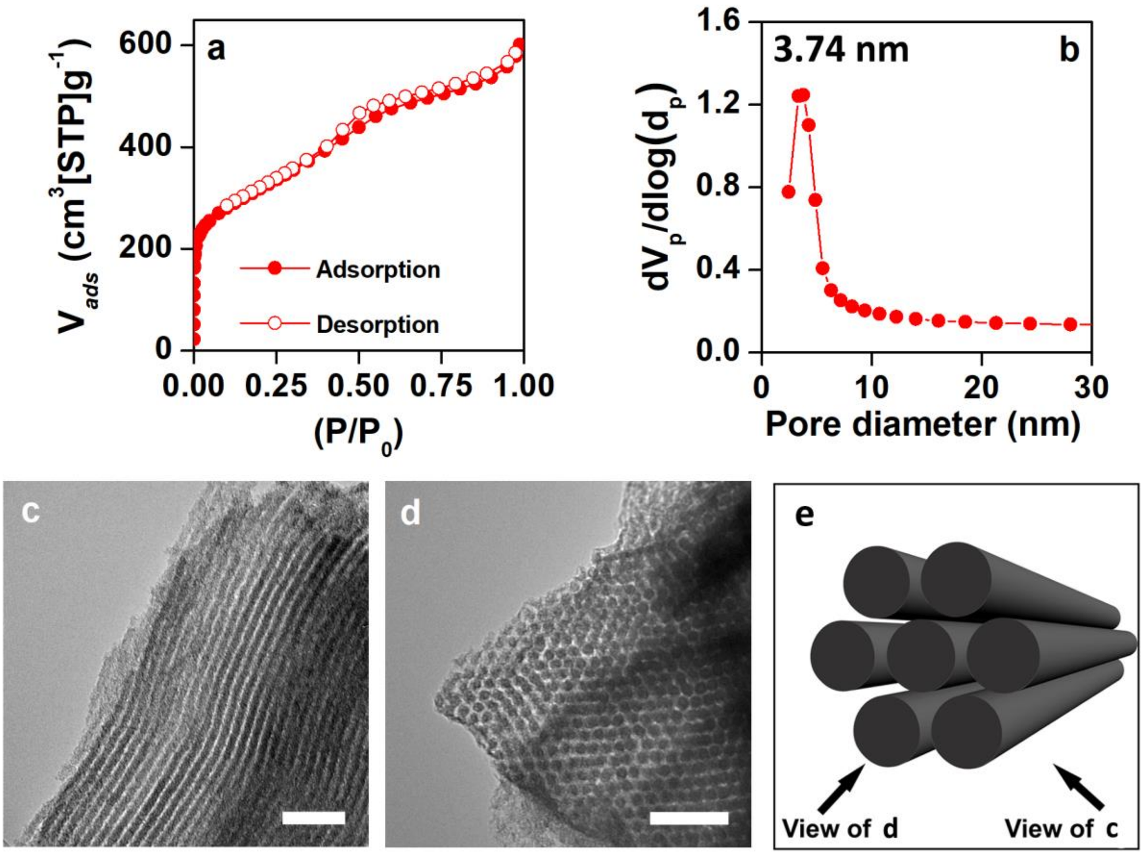

Supplementary Figure 1 Characterization of as-prepared CMK-3 structure. **a** N_2 adsorption/desorption isotherm and **b** Barrett-Joyner-Halenda (BJH) pore-size distribution. **c-d** TEM images and **e** illustration of hexagonally ordered mesoporous channels. The scale bars in c and d indicate 50 nm. The arrows in **e** indicate the point of view for CMK-3, which corresponds to TEM images of **c** and **d**.

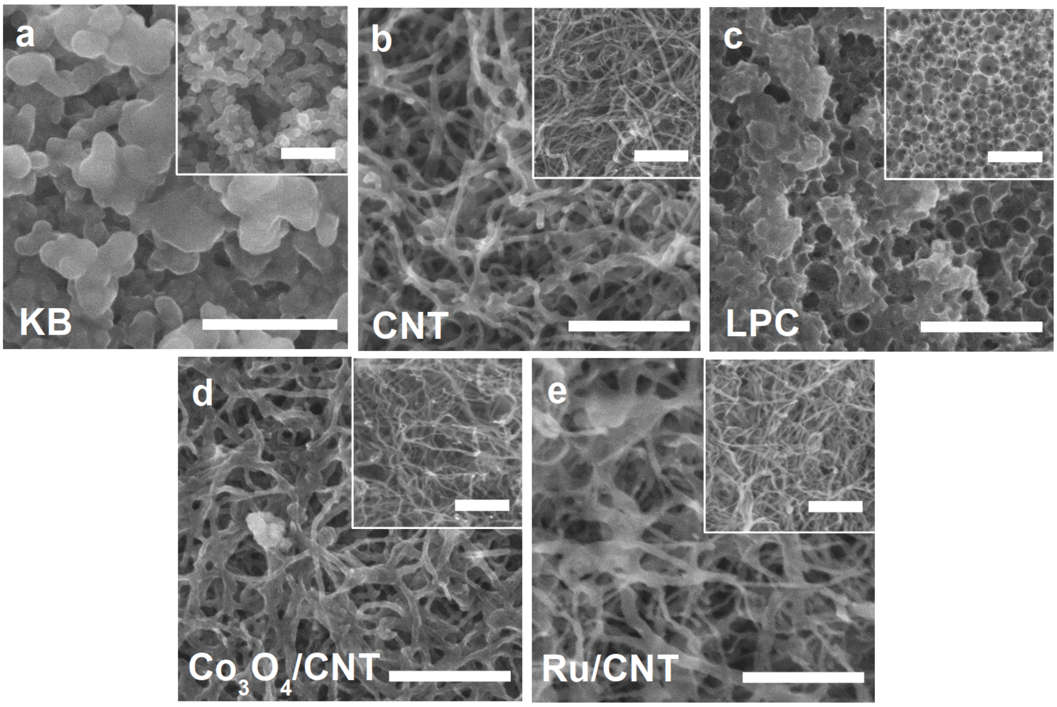

Supplementary Figure 2 Scanning electron microscopy (SEM) images for **a** KB, **b** CNT, **c** LPC, **d** Co_3O_4/CNT and **e** Ru/CNT electrodes after DC to 1.5 mAh. All the scale bars indicate 500 nm. The insets show the corresponding as-prepared electrodes and the scale bars indicate 250 nm.

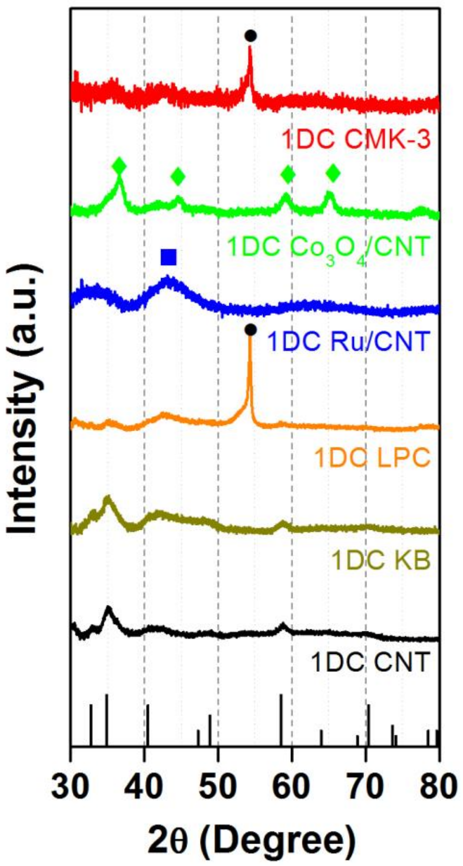

Supplementary Figure 3 X-ray diffraction (XRD) patterns of DC electrodes to 1.5 mAh and reference of Li_2O_2 (bottom line). The symbols of black circle (●), green diamond (♦) and blue square (■) indicate graphite (004) arising from porous carbon paper used as current collector for CMK-3 and LPC, face-centered cubic Co_3O_4 (fcc, *Fd3m*, JCPDS card no. 01-080-1533) and broad Ru reflection (JCPDS card No. 06-0663) respectively. The crystalline Li_2O_2 reflections appear for the 1DC LPC, KB and CNT.

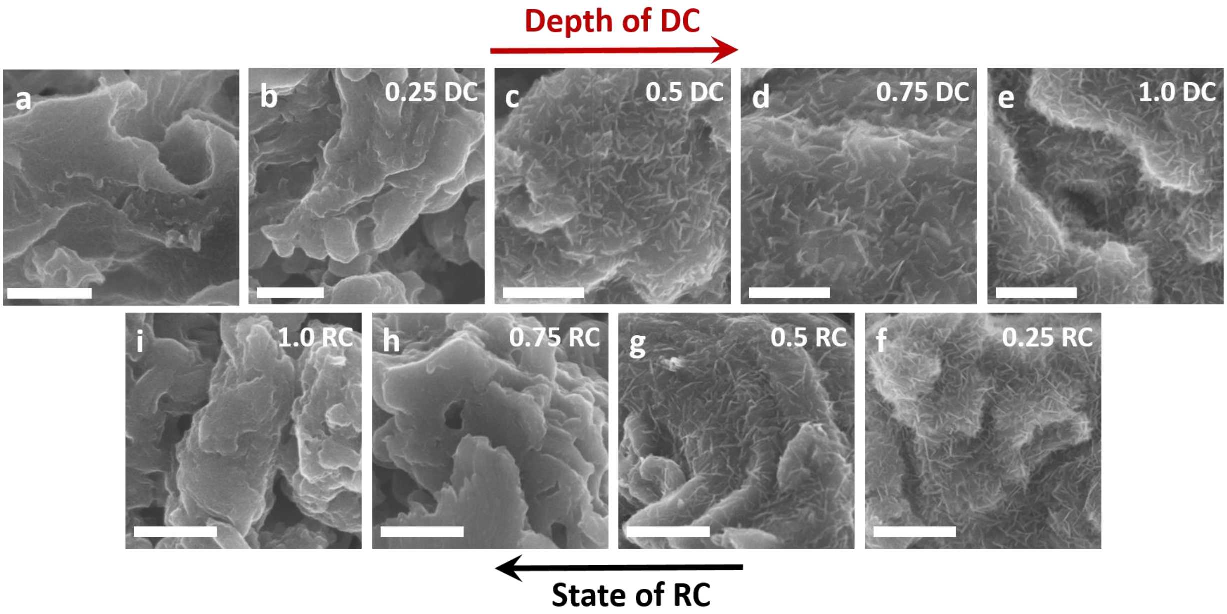

Supplementary Figure 4 SEM images of CMK-3 electrodes of **a** as prepared, **b–e** different depth of DC and **f–i** different state of RC denoted by Q/Q_{total} at a fixed Q_{total} of 1.0 mAh and the current rate of 50 mA g^{-1}_{carbon}. The scale bars indicate 500 nm.

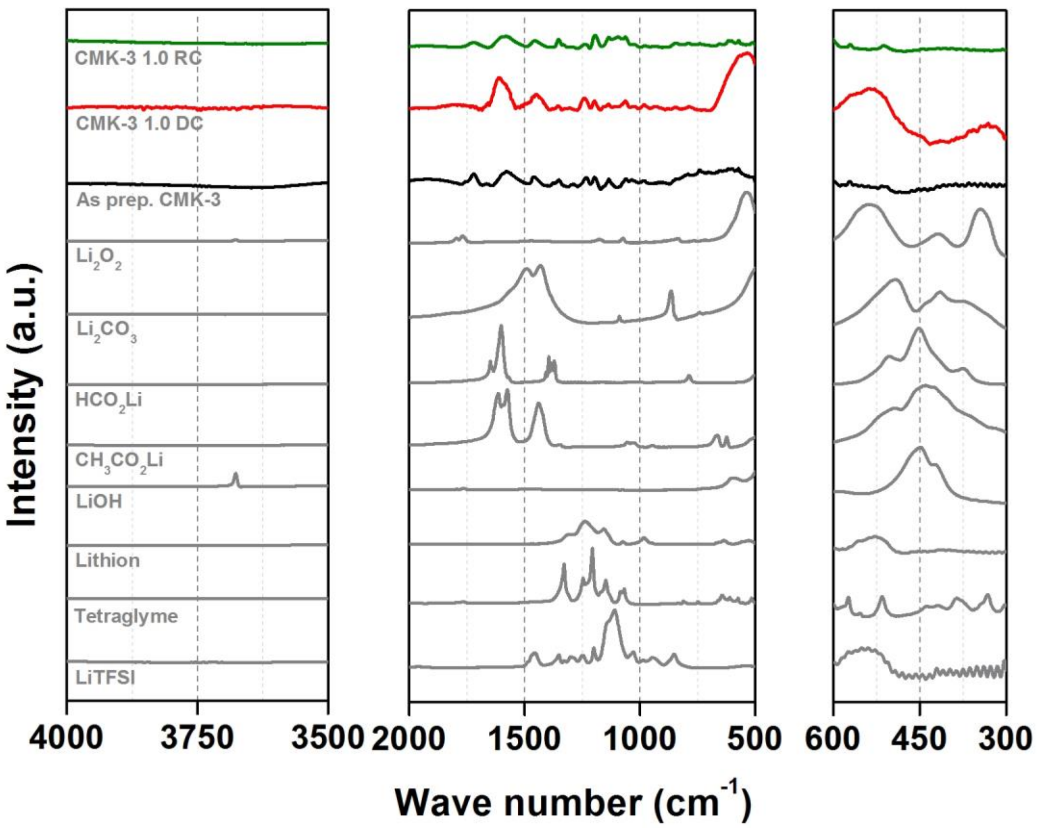

Supplementary Figure 5 FT-IR spectra of DC and RC CMK-3 electrodes with standard references of Li_2O_2, Li_2CO_3, HCO_2Li, CH_3CO_2Li, LiOH, LITHion (binder), tetraglyme and LiTFSI. All CMK-3 electrodes were washed with acetonitrile and dried at 60 ^{o}C in vacuum before measurement of FT-IR.1DC and 1RC samples were the electrodes after 100% DC and RC (a capacity of 1.0 mAh). The Li_2O_2-related signals at ~350 and ~535 cm^{-1} appear to 1DC CMK-3 and disappear after RC. The CO_3^{2-} stretching modes (~1440 and 1500 cm^{-1}) and CO_2^- stretching mode and deformation (1370–1615 cm^{-1}) are apparent for 1DC CMK-3[1].

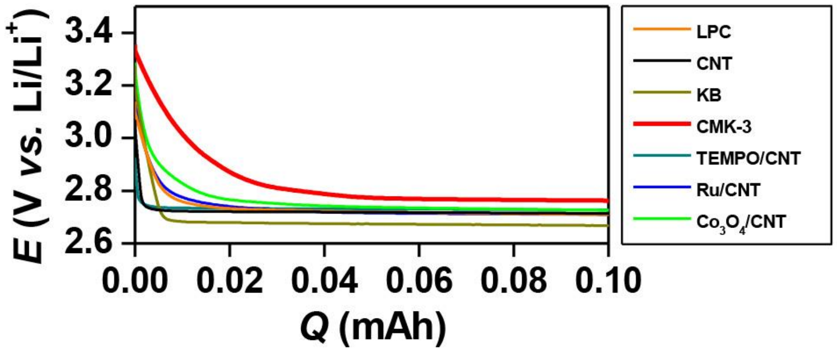

Supplementary Figure 6 Comparison of DC potentials for various carbon electrodes at initial stage (0–0.1 mAh), captured from Figure 1a. CMK-3 has higher DC potential than others.

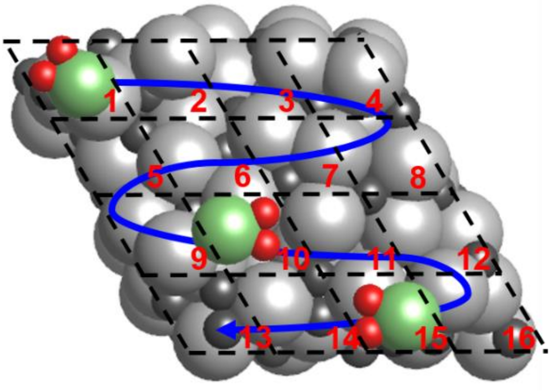

Supplementary Figure 7 The amorphous Li_2O_2 model used in this study[2]. The numbers indicate 16 sites for possible LiO_2 adsorption. Lithium is light gray and light green; Oxygen is dark gray and red.

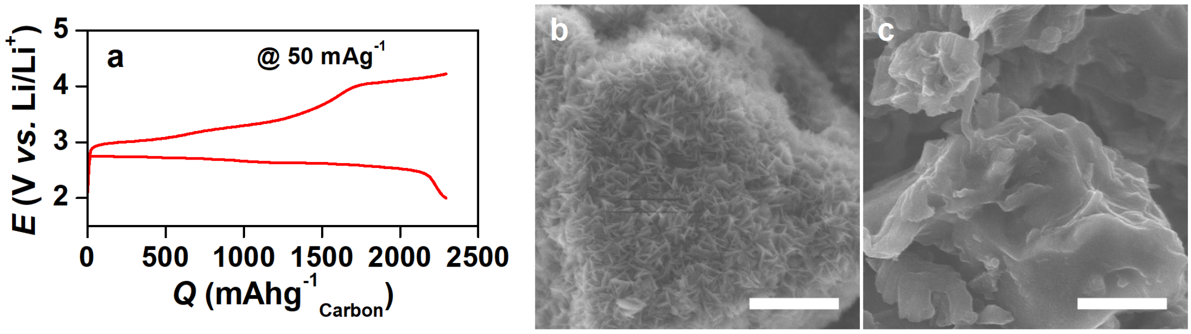

Supplementary Figure 8 Full DC and RC profiles of CMK-3 at the cut off DC potential of 2.0 V and RC to the equivalent capacity and electrode images. **a** Galvanostatic profile at a current rate of 50 mA g^{-1} $_{carbon}$. SEM images after **b** full DC and **c** full RC. The scale bars indicate 1 μm.

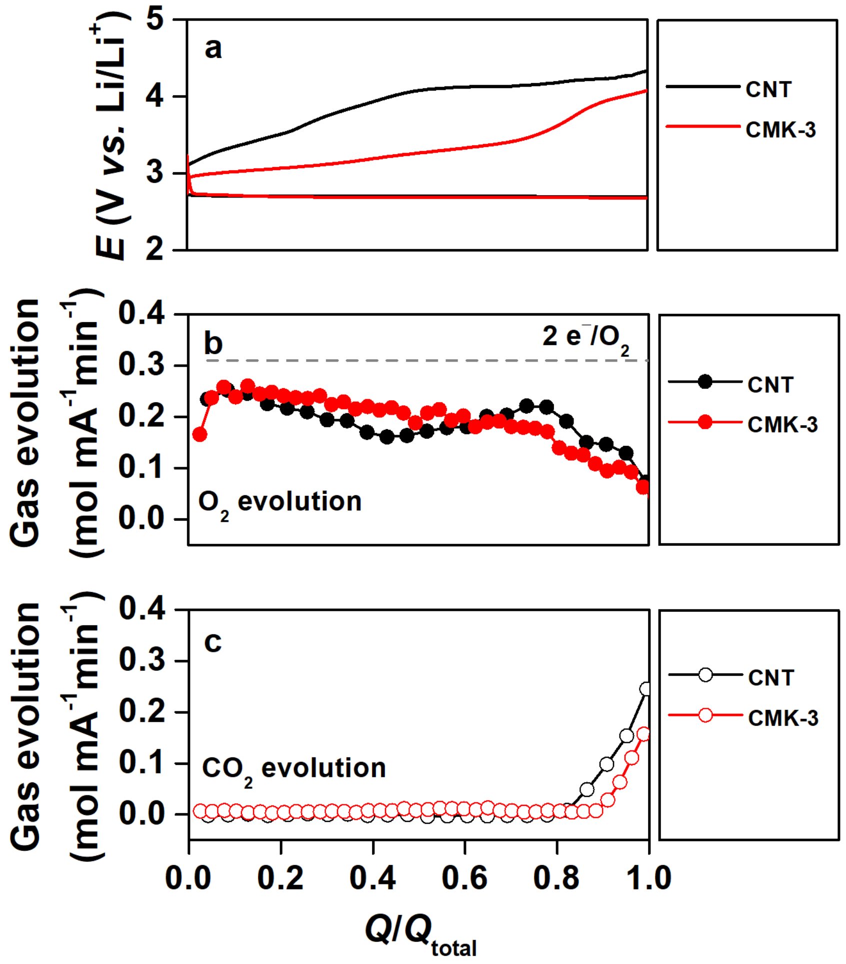

Supplementary Figure 9 Comparison of online electrochemical mass spectrometry (OEMS) data of CMK-3 and CNT electrodes under identical conditions. **a** DC-RC profiles. Online **b** O_2 and **c** CO_2 evolution data during RC. CMK-3 shows 3.17 e^-/O_2 oxidation during charge whereas the value of the same for CNT electrode is 3.31 e^-/O_2. It is further notable that the charging overpotential for CNT is much higher, leading to larger amount of CO_2 evolution.

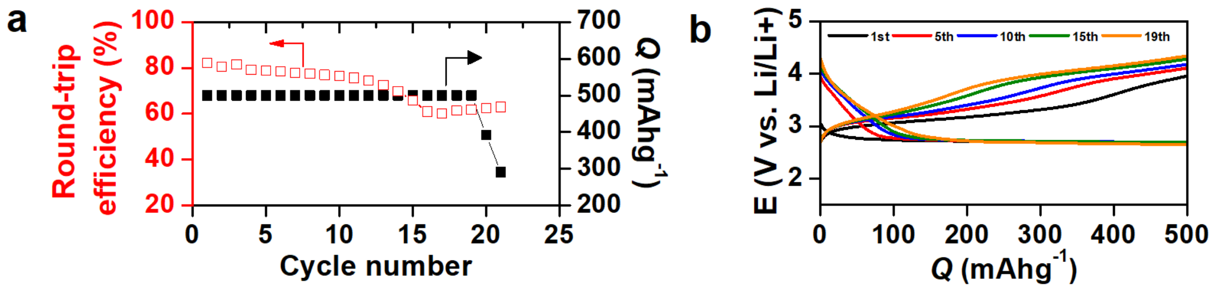

Supplementary Figure 10 Cycling performance of CMK-3. **a** The round-trip efficiency and **b** DC-RC curves at a limited capacity of 500 mAh g^{-1}_{carbon} and current rate of 50 mA g^{-1}_{carbon}.

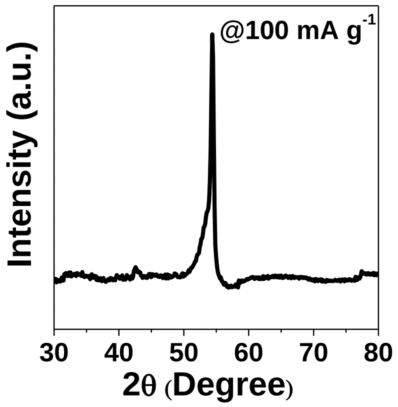

Supplementary Figurer 11 XRD patterns of DC product of conformal film acquired at a current rate of 100 mA g^{-1}_{carbon} and a fixed DC capacity of 500 mAh g^{-1}_{carbon}.

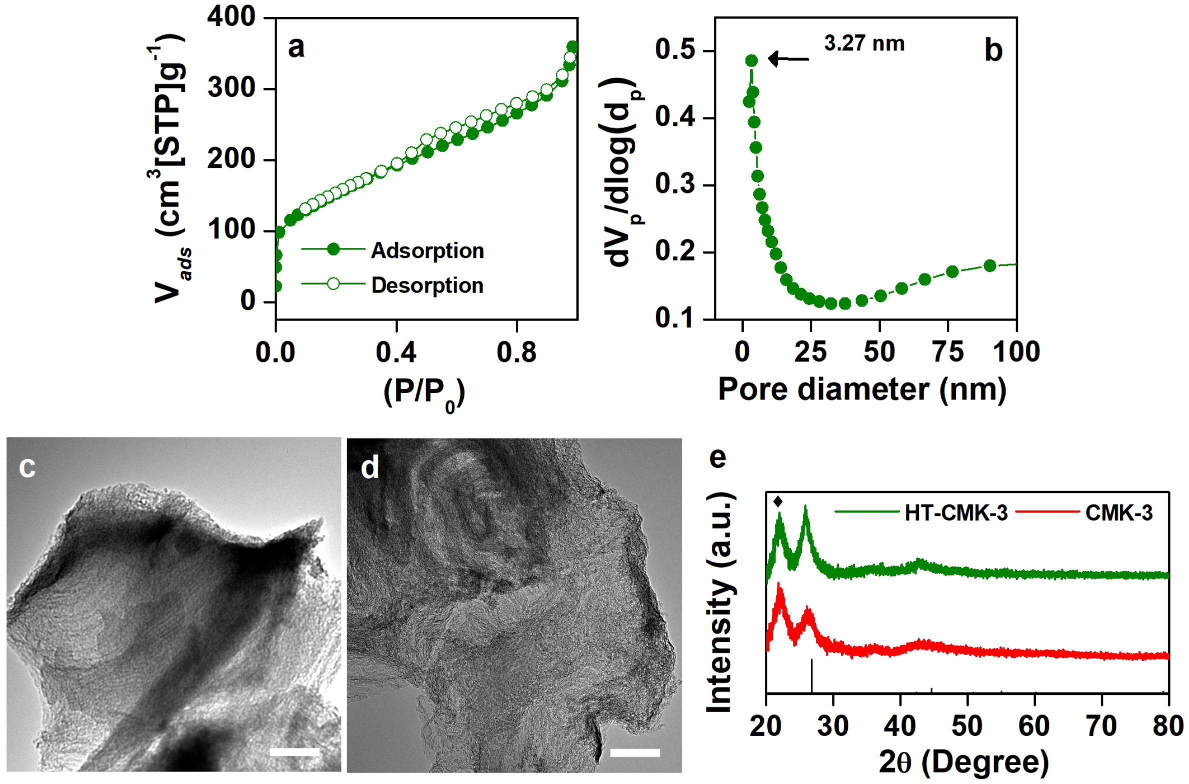

Supplementary Figurer 12 Characterizations of high-temperature annealed CMK-3 (HT-CMK-3). **a** N_2 adsorption/desorption isotherm, **b** BJH pore-size distribution, **c–d** TEM images of HT-CMK-3. The scale bars indicate 100 nm. **e** XRD data for as-prepared HT-CMK-3 (green, top) and CMK-3 (red, bottom) with the reference of graphite 002 ($2\theta = 26.7^o$) and 101 reflection ($2\theta = 44.6^o$) in the bottom line[3]. The increasing intensity and decreasing FWHM (full width at half maximum) of 002 reflection indicate more ordering in turbostratic carbon by annealing[4] but along with the disordered and clogged mesoporous channels in TEM images. The (♦) peak at $2\theta = 22^o$ comes from the Kapton tape used to cover the samples.

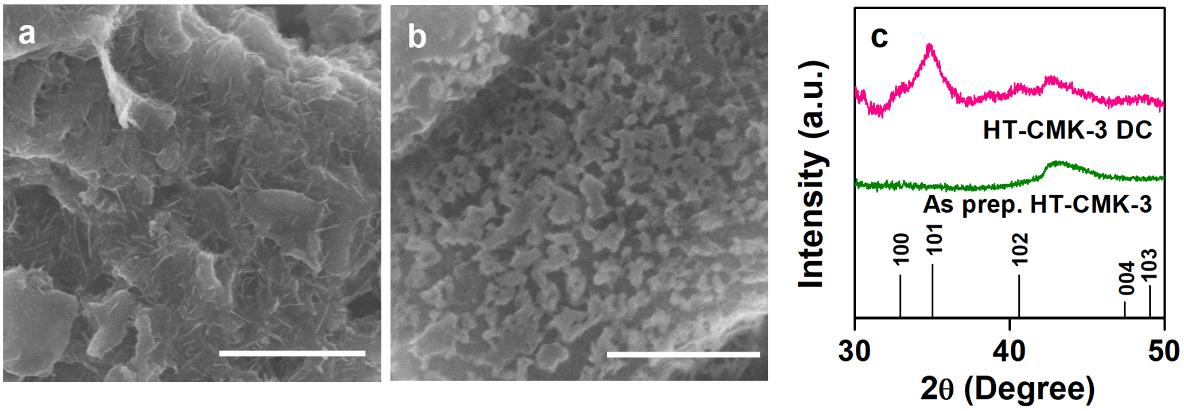

Supplementary Figure 13 SEM image and XRD result of HT-CMK-3 after DC to the fixed capacity of 1 mAh and a current rate of 50 mA $g^{-1}{}_{carbon}$. **a–b** SEM images in different areas of 1DC with **a** flake-shaped products and **b** lumps. The scale bars in **a** and **b** represent 1 μm and 500 nm respectively. **c** XRD patterns for as-prepared HT-CMK-3 (green) and after 1DC (pink) with the standard reference of Li_2O_2 (bottom line). The crystalline Li_2O_2 reflections clearly appear after DC.

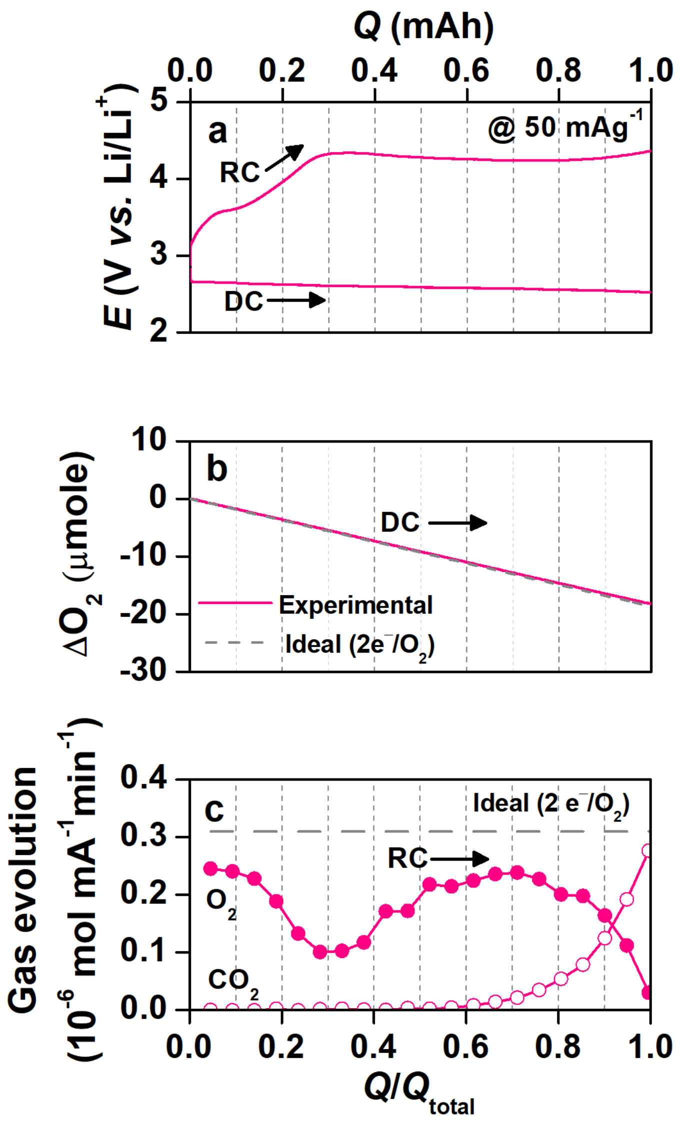

Supplementary Figure 14 *In situ* gas analysis for HT-CMK-3. **a** DC-RC curve at current rate of 50 mA $g^{-1}{}_{carbon}$ and limited capacity of 1.0 mAh. **b** The gas pressure with the corresponding DC (overall avg. 2.04 e^{-}/O_2). **c** O_2 gas (overall avg. 3.30 e^{-}/O_2) and CO_2 gas (over 0.6RC) evolution with the corresponding RC. The dashed line represents the ideal 2 e^{-}/O_2.

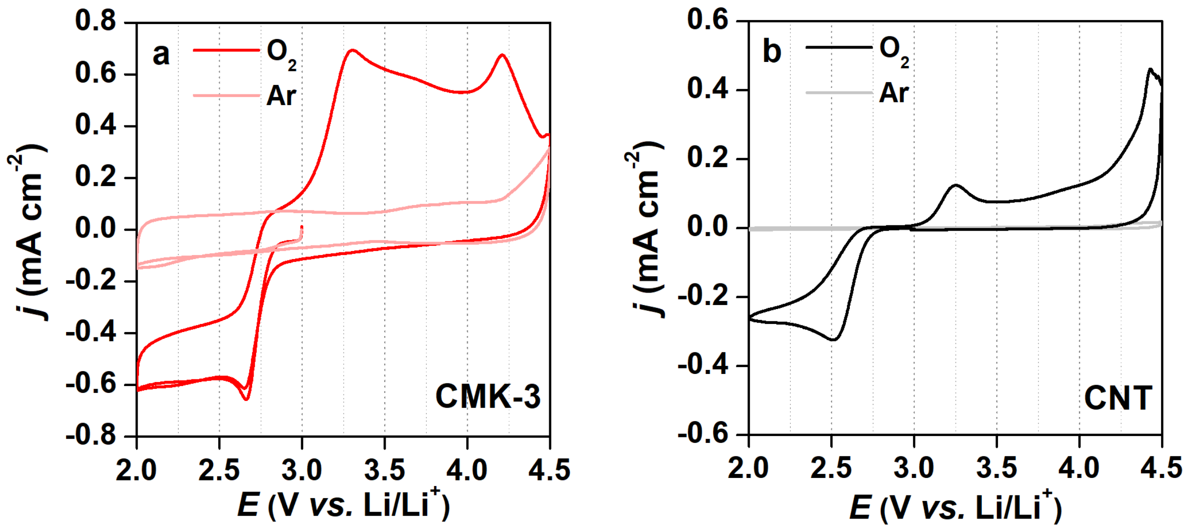

Supplementary Figure 15 CV curves same as Figure 5 but with *j* vs. *E* format for **a** CMK-3 and **b** CNT at a potential sweeping rate of 1 mV s^{-1}.

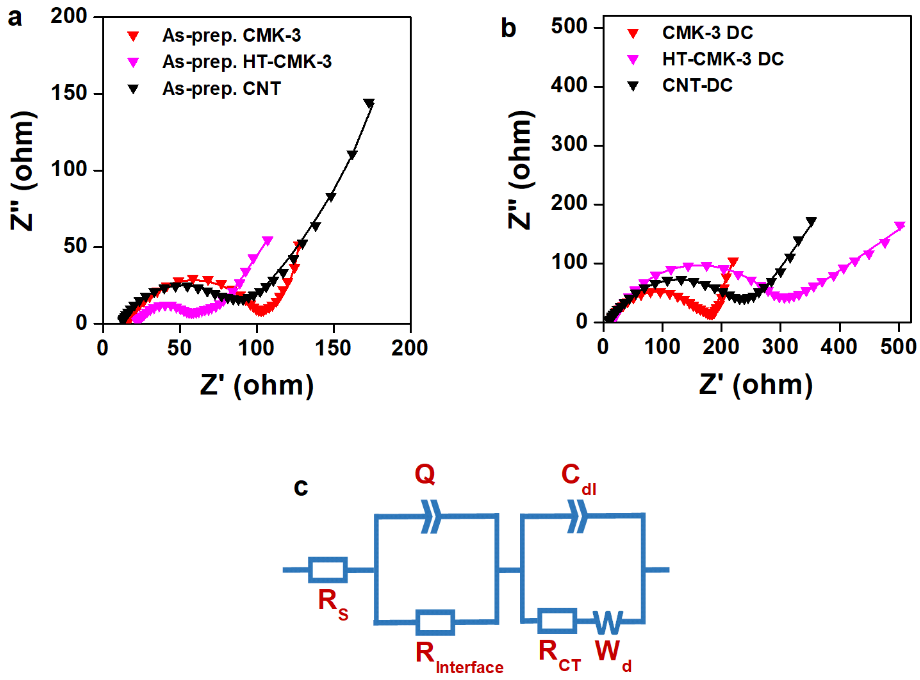

Supplementary Figure 16 Electrochemical impedance spectroscopy (EIS) analysis of **a** as-prepared and **b** 1DC CMK-3, HT-CMK-3 and CNT electrodes. The 1DC was examined at a fixed capacity of 1.0 mAh and a constant current of 50 mA g^{-1}_{carbon}.The dotted symbols and solid lines denote experimental data and simulated curves according to **c** the equivalent circuit, respectively. The R_S includes the ohmic resistance of electrolyte solution, and the electronic resistances of current collectors and metallic Li electrode. The $R_{interface}$ and Q denote the resistance and constant phase element of an interface layer, likely a cathode electrolyte interface (CEI) layer[5], respectively. The R_{CT} represents the charge-transfer resistance of the surface layer on the carbon electrode in parallel with its double layer capacitance, C_{dl}. The W_d is the diffusion related factors of Li^+ ion.

The $R_{interface}$ and R_{CT} values are significantly affected from Li_2O_2 after DC. From the simulated curves, the sum of $R_{Interface}$ and R_{CT} for as-prepared electrodes is estimated to 84 Ω for CMK-3, 45 Ω for HT-CMK-3 and 78 Ω for CNT. It is apparent that HT-CMK-3 and CNT electrode have lower resistance than CMK-3 electrode. After DC, the sum of $R_{Interface}$ and R_{CT} is increased to 170 Ω for CMK-3, 301 Ω for HT-CMK-3 and 220 Ω for CNT, demonstrating the lowest resistance of ultrathin and amorphous Li_2O_2 from CMK-3 and the highest one of crystalline and lump-shape Li_2O_2 from HT-CMK-3.

Supplementary Table 1 Total surface area (SA_{total}), volume (V_{total}), diameters of mesopores (d_{meso}), micropores (d_{micro}) and volume for mesopore (V_{meso}) and micropore (V_{micro}) for CMK-3 and high temperature-annealing CMK-3 (hereon denoted as HT-CMK-3). The diameters of meso- and micropores are similar to CMK-3 and HT-CMK-3 whereas SA_{total} and V_{total} is reduced by almost half. O/C indicates atomic ratio of oxygen to carbon acquired from X-ray photoelectron spectroscopy (XPS). The atomic ratio of O is insignificant for both electrodes and its decreasing after high-temperature annealing does not significantly alter the wetting of electrode from tetraglyme electrolyte solution[6].

	**SA_{total} (m^2g^{-1})**	**d_{meso} (nm)**	**d_{micro} (nm)**	**V_{total} (cm^3g^{-1})**	**V_{meso} (cm^3g^{-1})**	**V_{micro} (cm^3g^{-1})**	**O/C**
**CMK-3**	1128	3.74	0.71	0.9308	0.6661	0.2647	0.06
**HT-CMK-3**	546	3.27	0.70	0.5557	0.4340	0.1217	0.03

Supplementary Table 2 Calculated DC and RC overpotentials for all sites in Supplementary Figure 7.

#	DC	RC
**1**	0.05	0.03
**2**	0.48	0.31
**3**	0.46	0.79
**4**	0.28	0.51
**5**	0.29	0.48
**6**	0.26	0.14
**7**	0.47	0.28
**8**	0.14	0.28
**9**	0.33	0.36
**10**	0.47	0.40
**11**	0.69	1.07
**12**	0.53	0.45
**13**	0.23	0.20
**14**	0.43	0.40
**15**	0.57	0.84
**16**	0.30	0.26
**$(1\bar{1}00)$ Li_2O_2**	0.52	0.86

Supplementary References

1 Hong, M., Choi, H. C. & Byon, H. R. Nanoporous NiO plates with a unique role for promoted oxidation of carbonate and carboxylate species in the Li–O_2 battery. *Chem. Mater.* **27**, 2234–2241 (2015).

2 Tian, F., Radin, M. D. & Siegel, D. J. Enhanced charge transport in amorphous Li_2O_2. *Chem. Mater.* **26**, 2952–2959 (2014).

3 Li, Z. Q., Lu, C. J., Xia, Z. P., Zhou, Y. & Luo, Z. X-ray diffraction patterns of graphite and turbostratic carbon. *Carbon* **45**, 1686–1695 (2007).

4 Inagaki, M., Toyoda, M. & Tsumura, T. Control of crystalline structure of porous carbons. *RSC Adv.* **4**, 41411–41424 (2014).

5 Bardenhagen, I. *et al.* In situ investigation of pore clogging during discharge of a Li/O_2 battery by electrochemical impedance spectroscopy. *J. Power Sources* **278**, 255–264 (2015).

6 Wong, R. A. *et al.* Structurally tuning Li_2O_2 by controlling the surface properties of carbon electrodes: Implications for Li–O_2 batteries. *Chem. Mater.* **28**, 8006–8015 (2016).
